# Optical Fiber Grating Hydrogen Sensors: A Review

**DOI:** 10.3390/s17030577

**Published:** 2017-03-12

**Authors:** Jixiang Dai, Li Zhu, Gaopeng Wang, Feng Xiang, Yuhuan Qin, Min Wang, Minghong Yang

**Affiliations:** 1 National Engineering Laboratory for Fiber Optic Sensing Technology, Wuhan University of Technology, Wuhan 430070, China; djx409081947@163.com (J.D.); zhuli1203@whut.edu.cn (L.Z.); wanggaopeng2003@hotmail.com (G.W.); fengxiang@whut.edu.cn (F.X.); qinyuhuan@whut.edu.cn (Y.Q.); bluebluecherry@163.com (M.W.); 2 Key Laboratory of Fiber Optic Sensing Technology and Information Processing, Ministry of Education, Wuhan University of Technology, Wuhan 430070, China

**Keywords:** hydrogen sensor, optical fiber grating, sensitive materials

## Abstract

In terms of hydrogen sensing and detection, optical fiber hydrogen sensors have been a research issue due to their intrinsic safety and good anti-electromagnetic interference. Among these sensors, hydrogen sensors consisting of fiber grating coated with sensitive materials have attracted intensive research interests due to their good reliability and distributed measurements. This review paper mainly focuses on optical fiber hydrogen sensors associated with fiber gratings and various materials. Their configurations and sensing performances proposed by different groups worldwide are reviewed, compared and discussed in this paper. Meanwhile, the challenges for fiber grating hydrogen sensors are also addressed.

## 1. Introduction

With aggravated air pollution and increasing fossil fuel consumption, exploiting clean and renewable energy is urgent for humankind. Hydrogen, which is an ideal candidate as a future energy source, has attracted much research interest [1,2,3] due to its recyclable and non-polluting characteristics. However, hydrogen is dangerous due to its high diffusivity and flammability, and its explosive limit covers a wide range of H_2_-air mixtures (4%–75% (*v/v*)). Commercial electrochemical hydrogen sensors still have the potential for explosion due to potential electric sparks. Optical fiber hydrogen sensors can be intrinsically safe as they employ optical signals as a sensing medium, and as such they have attracted much research interest. Several kinds of optical fiber hydrogen sensors, such as evanescent sensors [4,5,6], micro-mirror sensors [7,8], interference sensors [9], surface plasmon resonance (SPR) sensors [10], acoustic resonator sensors [11], and fiber grating sensors [12,13,14,15,16,17,18,19,20,21,22,23,24,25,26,27,28,29,30,31,32,33,34,35], have been proposed in recent years. Compared to other optical fiber hydrogen sensors, hydrogen sensors based on fiber grating are more suitable for distributed measurement and temperature compensation because of their wavelength multiplexing capability. Several review articles [36,37,38,39] related to optical hydrogen sensors have been published. These papers have summarized optical fiber hydrogen sensors from different perspectives. In this paper, a review paper about optical fiber grating hydrogen sensors is presented from a distinct perspective.

Since hydrogen molecules cannot be detected by the spectral absorption method, utilizing materials that can react with hydrogen is an essential method for hydrogen sensing technology. Hydrogen-sensitive materials are indispensable for preparing hydrogen sensing probes. According to classification of hydrogen-sensitive materials, two types of fiber grating hydrogen sensors have been reported. The first kind of fiber grating hydrogen sensor is based on Pd [12,13,14,15,16,17,18,19,20] or Pd alloys [21,22,23,24,25], which can produce volume expansion or optical constant change during hydrogen response. The other type is based on Pt-loaded WO_3_ coatings [40,41,42,43,44] or other oxide materials [45] undergoing an exothermic or gasochromic reaction [46,47] in a hydrogen atmosphere. The configurations and performances of these sensors are presented in the following paragraphs.

## 2. Optical Fiber Grating Hydrogen Sensor Based on Pd and Pd Alloy

**Figure 1:**
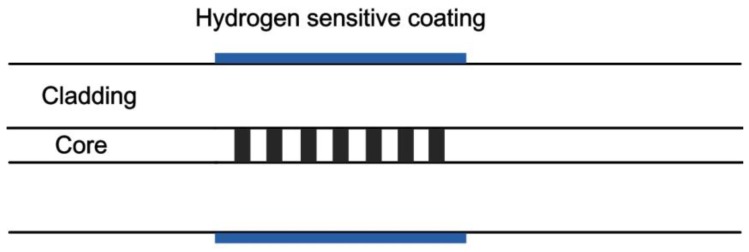
Typical structure of a fiber Bragg grating (FBG) hydrogen sensor [12].

In 1999, the first Pd-based fiber Bragg grating (FBG) hydrogen sensor was proposed by Sutapun et al. [12]. The typical structure of the sensor is shown in Figure 1. FBG with cladding of 35 μm was evaporated with 560 nm of pure Pd film for hydrogen characterization. The sensor showed linear wavelength shift when the hydrogen concentration (in N_2_ atmosphere) ranged from 0.3% (*v/v*) to 1.8%. When the hydrogen concentration was over 1.8%, the hydrogen sensor deteriorated and became irreversible. The main reason for this phenomenon is due to the poor stability of pure Pd film. Additionally, the stress caused by Pd film is about three times greater than that of bulk Pd when the hydrogen concentration is between 0.5% and 1.4% at room temperature, which is a very interesting phenomenon.

During the same year, Tang et al. [13] reported a FBG hydrogen sensor based on a Pd tube. By employing a Pd tube (with different thickness) as the hydrogen-sensitive material, the sensitivity of the FBG hydrogen sensor was increased. However, the response time of the sensor (under 4% H_2_ in N_2_ atmosphere) was more than 200 min at room temperature. The influence of operating temperature on FBG hydrogen sensors was also investigated in this paper. When the temperature was between 23 °C and 45 °C, the hydrogen sensor displayed the largest wavelength shift. However, the response time of the sensor was still too long. By increasing the operating temperature to 95 °C, the response time can be less than 2 min. However, the sensitivity of the hydrogen sensor will be sacrificed due to the lower hydrogen absorption capability of the Pd tubes at higher temperature. The purging process under N_2_ and air was also studied in this paper, and quicker degassing rate was observed in air. This result implies that atmosphere also has significant influence on the performance of the sensor.

A Pd-based FBG hydrogen sensor was developed by Zalvidea et al. [14] in 2004. Pd film was coated on tapered single mode fiber, and FBG was used to obtain the optical power reflected by the sensing probe. This configuration enables double interaction of light with the deposited Pd film, and therefore the sensitivity of the sensor can be increased.

In 2007, a 25-μm FBG coated with 5 nm of Pd was proposed for hydrogen detection by Aleixandre et al. [15]. The response time of the sensor was reduced within 10 min. Compared to the previous work, the repeatability of the sensor was obviously improved. However, the hydrogen sensor showed a longer response time due to the superficial oxidation of Pd film.

To improve the performance of hydrogen sensors, optical heating method was utilized by Buric et al. [16]. FBG was written into double-clad fiber, followed by sputtering glue metal and 150 or 500 nm of Pd film. With heating power of 560 mW (910 nm diode laser), the hydrogen sensor showed a quick response rate (less than 10 s). Meanwhile, the hydrogen response was repeatable, and there was little hysteresis at room temperature and lower temperature of −50 °C. This work exhibited an effective approach to prepare Pd-based FBG hydrogen with a quick response rate and high sensitivity, especially at low temperatures. In 2009, further improvement was achieved by inscribing FBG in 1-cm-high attenuation fibers and then splicing with single-mode fiber [17]. The heating efficiency was greatly improved by this technique, which enabled the detection of 1% hydrogen at −150 °C. This method can provide multiplexing sensing ability due to the lower optical power loss only at the Bragg grating section and the spicing junction. Moreover, there was little hysteretic effect even when the thickness of the Pd film was between 150 and 500 nm.

**Figure 2:**
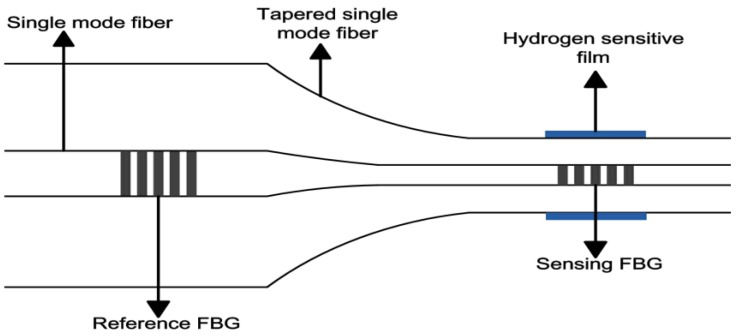
FBG hydrogen sensor based on a tapered single-mode fiber [18].

Another way to increase the sensitivity of FBG hydrogen sensor is using by an FBG with a smaller diameter. As shown in Figure 2, the sensing probe was fabricated by depositing 150 nm Pd on tapered FBG with diameter of 50 μm [18]. The proposed sensing head was able to detect H_2_ concentration in the range of 0.1%–1% (*v/v*) in N_2_, and sensitivity of 81.8 pm was attained at room temperature. However, pure Pd film still has the possibility to peel off due to its poor adhesion towards optical fiber.

The poor adhesion between thick Pd film and optical fiber can be improved by adding a polymer coating as an intermediate layer [19,20]. The characterization of these hydrogen sensors was performed in power transformer oil. By sputtering 20 nm Ti and 560 nm Pd film on FBG coated with polyimide layer, the sensitivity of the sensor can reach to 13.5 ppm/pm when set in transformer oil. The polymer layer can successfully prevent the blistering of Pd film, resulting in a significant improvement of the reproducibility of Pd-based hydrogen sensors. The alternative way to improve the stability of FBG hydrogen sensors is adopting Pd alloys. In 2012, Dai et al. [21] proposed an FBG hydrogen sensor based on Pd_91_Ni_9_ composite film. FBG with a diameter of 17 μm was prepared by removing fiber cladding in HF solution. Then, Pd_91_Ni_9_ composite film was deposited on the side-face of etched FBG. X-ray powder diffraction (XRD) results proved Pd_91_Ni_9_ composite film had good structural stability during hydrogen response. At room temperature of 23 °C, the sensor showed an approximately 15 pm wavelength shift towards 1% hydrogen in air, and the response time was no more than 5 min. Moreover, the hydrogen sensor displayed good repeatability. In the next year, Pd_76_Ag_24_ composite film was employed for hydrogen sensing [22]. Although the FBG (diameter of 20.6 μm) hydrogen sensor showed good repeatability, the sensitivity was only about 10 pm/1% (*v/v*) in air. Two years later, the sensitivity of the hydrogen sensor was greatly increased by employing polypropylene sheet as a flexible substrate [23]. Wavelength shifts of 37 pm under 1% hydrogen were observed in this paper; the better sensitivity of the sensor was mainly due to the much lower Young’s modulus of the polymer sheet. Although the hydrogen sensor still has good response towards 4% hydrogen, the response time nearly doubled due to the oxidation of the composite film after setting in air for six months. The oxidation of hydrogen sensitive film not only has a negative effect on the response rate, but also can reduce its hydrogen responsibility. Therefore, the detection threshold of the hydrogen sensor can be increased due to this passive effect.

In some special facilities, there is little oxygen and an FBG hydrogen sensor based on a Pd alloy has better potential applications. In 2015, FBG hydrogen sensor based on Pd/Ag composite film displayed better stability than pure Pd film [24] in transformer oil. When the operating temperature increased from 20 °C to 80 °C, the Pd/Ag-based hydrogen sensor showed lower sensitivity and a quicker response rate. A similar comparison was conducted in 2016 [25]. An FBG hydrogen sensor based on Pd and Pd_58_Cr_42_ was reported for hydrogen concentration monitoring in the same facilities. After depositing TiO_2_ as an intermediate layer, the hydrogen-sensitive films were coated on FBG. These sensors displayed nearly 15 pm wavelength shifts when the hydrogen concentration increased to 650 ppm. The relatively low sensitivity can be attributed to the employment of standard FBG in this work. The Pd-based sensor had quicker response rate and higher sensitivity, while Pd_58_Cr_42_ showed the capability to detect higher concentration hydrogen. And these experimental results proved that Pd_52_Cr_48_ can enhance the dissolved hydrogen detection range in transformer oil.

Another type FBG hydrogen sensor, which was based on optical constant change of Pd film when exposed to hydrogen, was demonstrated by Tien et al. [26] in 2008. FBG was glued on a silicon V groove and polished with residual diameter of 62.9 μm. Thus, the central wavelength of FBG is sensitive to the change of the ambient refractive index. A 20-nm Pd film was deposited on the polished FBG. In contrast to the red wavelength shift of the sensor based on mechanical stress caused by volume expansion, the proposed sensing device displayed blue wavelength shifts during the hydrogen response process. The main reason for this phenomenon is that the influence of decreased refractive index is greater than that of imposed stress. Since the FBG was fixed on v-groove, the change of grating period was greatly suppressed. Thus, effective refractive index of Bragg grating can be remarkably reduced by the volume expansion of Pd film, leading to the decrease of the central wavelength.

**Figure 3:**
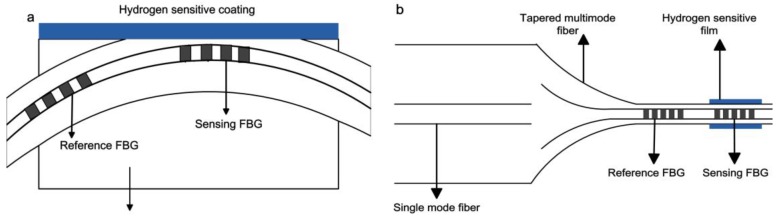
(**a**) Side-polished FBG (SPFBG) hydrogen sensor fixed on quartz substrate [27]; (**b**) Micro-FBG (MFBG) hydrogen sensor based on tapered multimode fiber [28].

In 2009, Schroeder et al. [27] proposed a side-polished FBG hydrogen sensor based on evanescent-interaction of Pd film. Two Bragg gratings with lengths of 1.5 mm and 2 mm were cemented in the groove of glass block. The FBG (1.5 mm) was polished and coated with 50 nm Pd (in Figure 3a). In contrast to the above work [26], the transverse magnetic (TM) mode of emitted light was used for hydrogen characterization. The hydrogen sensor showed a nonlinear response toward to hydrogen from 0.1% to 5% (*v/v*) in Ar atmosphere. This phenomenon was more obvious during the exposure of 1% and 2%, which could be due to α-ß phase transition of Pd film. The response time for 1% hydrogen was about 2 min, while that for 4% hydrogen was about 30 s. Although the proposed device was much more complex, a higher sensitivity can be obtained by this work. Another type of FBG hydrogen based on this principle was reported in 2015 [28]. A multimode fiber (62.5/125 μm) was spliced to single mode fiber, and then tapered with the aid of a 2-mm flame. A 193-nm excimer laser was utilized to write 3-mm grating in the tapered multimode fiber, and 15 nm of Pd was deposited on the grating section as a sensitive layer (in Figure 3b). The proposed sensor (diameter of 3.3 μm) displayed −1.08 nm towards 5% hydrogen (in nitrogen atmosphere), with a response time of 60 s at room temperature.

**Figure 4:**
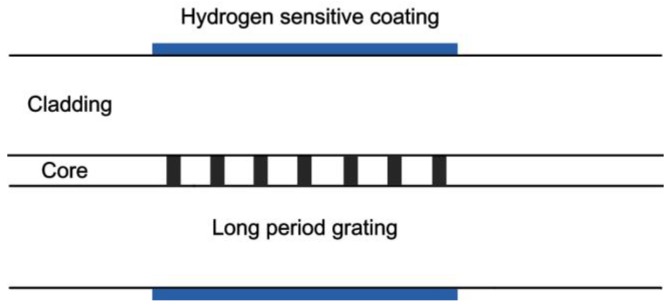
Typical structure of a long-period fiber grating (LPFG) hydrogen sensor [29].

Long period fiber grating (LPFG) is sensitive to external refractive index change without the side-polishing process. Therefore, it can be coated with hydrogen sensitive coating for hydrogen detection. The typical structure of the sensor is illustrated in Figure 4. In 2006, Trouillet et al. [29] compared the performance of FBG and LPFG coated with 50 nm Pd film. When the hydrogen concentration was set at 4%, the wavelength shifts of FBG and LPFG were approximately 14 pm and 7 nm (fundamental mode) respectively. The wavelength shift of the latter is nearly 500 times that of the former. After two years, the hydrogen-sensitive performance of LPFG coated with 70 nm of Pd was investigated by Wei et al. [30]. The resonance wavelength of LPFG decreased obviously as hydrogen concentration increased from 0% to 16% (in He atmosphere) at 30 °C, 100 °C, 150 °C and 200 °C, and the response time at all temperatures was less than 70 s. At an operating temperature of 30 °C, the hydrogen response of the sensor was especially remarkable. The hydrogen absorption capability of Pd decreased significantly as temperature increased, leading to a lower change in the refractive index of Pd film at higher temperatures. At the operating temperature of 30 °C, the sensor exhibited excellent sensitivity during hydrogen response, shifting more than 4 nm towards 4% H_2_. These Pd-coated LPFG and MFBG hydrogen sensors showed excellent sensitivity during hydrogen response, but a little hysterics effect still can be observed due to the phase transition of Pd film.

Side-polished FBG (SPFBG) has an interesting structure that is intrinsically sensitive to curvature [48], which can be utilized to improve the sensitivity of FBG hydrogen sensor. SPFBG was prepared by polishing common FBG with a motor-driven polishing wheel [49]. By sputtering the WO_3_-Pd composite film on side-polished FBG [31], the sensitivity and repeatability can be greatly improved.

**Figure 5:**
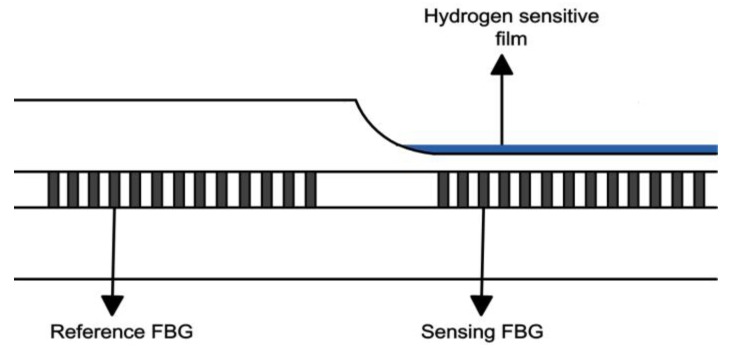
Typical structure of SPFBG hydrogen sensor [31].

The typical structure of the SPFBG hydrogen sensor is shown in Figure 5. Compared to standard FBG, SPFBG can increase the sensitivity of the sensor more than 100%. Similar results were also proposed by Jiang et al. [32]. In power transformer oil, SPFBG (with residual thickness of 20-μm cladding) coated with 560 nm of Pd/Ag composite film exhibited a sensitivity of 0.477 pm/(μL/L), which was 11.4 times higher than that of the common FBG hydrogen sensor. Subsequent improvements were reported in 2016 [33], and the hydrogen sensor showed a linear response when exposed to different concentrations of hydrogen at room temperature. All these results prove the FBG hydrogen sensor has great potential to monitor hydrogen concentration in a power transformer.

**Figure 6:**
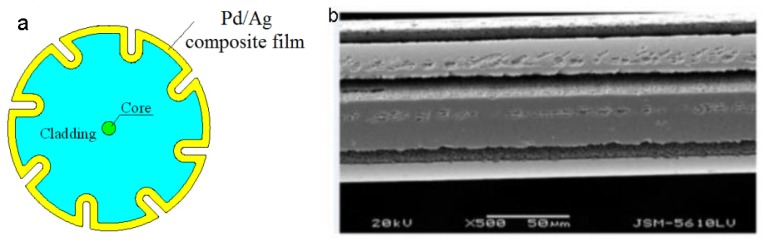
(**a**) Configuration of micro-machined FBG hydrogen sensor [34]; (**b**) SEM of micro-machined FBG coated with Pd/Ag composite film [34].

Recently, a novel technique to enhance the sensitivity FBG hydrogen was presented by Karanja et al. [34]. The cross section schematic diagram of the sensing probe is shown in Figure 6a. Fiber Bragg gratings were micro-machined by femtosecond laser to form microgrooves along the fiber, followed by sputtering 520 nm of Pd/Ag composite film on their grating section (Figure 6b). At room temperature (25 °C), the maximum sensitivity of the micro-machined FBG was increased more than 350% when compared with the standard FBG, which can be attributed to the larger surface area of fiber coated with Pd/Ag composite film. A similar phenomenon was observed when the ambient temperature was increased to 35 °C. Notably, the sensitivity the hydrogen sensor can be increased more 10 times at the higher operating temperature. For instance, the wavelength shift of 75-mW micro-machined FBG coated with 520 nm of Pd/Ag composite film was only 47 pm towards 4% hydrogen at 25 °C, while at 35 °C this was increased to nearly 700 pm. More attention should be concentrated on this phenomenon, which demonstrated that much higher sensitivity can be achieved by increasing the operating temperature of hydrogen-sensitive film.

**Figure 7:**
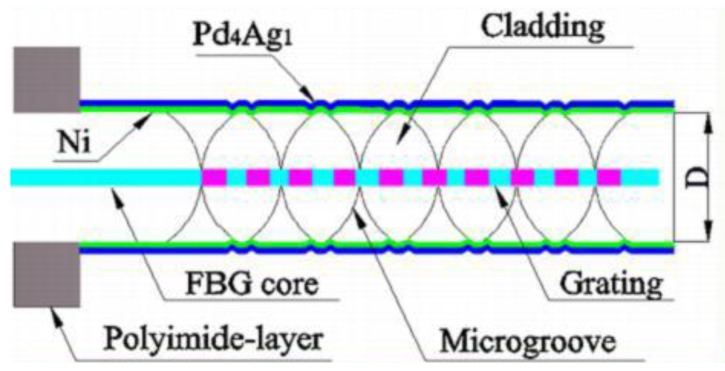
Configuration of double spiral FBG hydrogen sensor [35].

In the following year, FBG with a spiral groove sputtered with Pd/Ag film (in Figure 7), which was proposed by the same group, was used for hydrogen detection [35]. At room temperature, double spiral micro-structured FBG exhibits 51.5 pm/% responding to hydrogen in air, which is 7.5 times higher that of unprocessed FBG. In addition, the hydrogen sensor can detect 0.2% hydrogen in air. These results indicate the sensitivity of the sensor can be greatly improved by increasing the area for film deposition.

## 3. Optical Fiber Grating Hydrogen Sensor Based on Exothermic Reaction and Gasochromic Effect of Sensitive Materials

At present the fastest response FBG hydrogen sensor is based on Pt-loaded WO_3_ undergoing an exothermic reaction in hydrogen atmosphere [36,37]. The chemical reaction can be expressed as the following equation [40]:

(1) $${\mathrm{WO}}_{3}+{\mathrm{xH}}_{2}\overset{\mathrm{Pt}}{\to }{\mathrm{WO}}_{3-x}+{\mathrm{xH}}_{2}O$$

(2) $${\mathrm{WO}}_{3-x}+\frac{x}{2}O_{2}\overset{\mathrm{Pt}}{\to }{\mathrm{WO}}_{3}$$

WO_3_ can react with hydrogen drastically by utilizing Pt as a catalyst, which can release energy to the surrounding environment. By measuring the temperature change caused by the exothermic reaction, the hydrogen concentration can be calculated. When there is no hydrogen, WO_3−x_ can be oxidized to form WO_3_ in air. Therefore, the hydrogen sensor based on these reactions can be used for repeated measurements.

In 2007, Caucheteur et al. [40] proposed the first FBG hydrogen sensor based on this principle. FBGs with different lengths (0.5–4 cm) were used for hydrogen sensing. FBGs with a length of 4 cm had the lowest detecting threshold of 1% hydrogen in air, which could be due to the more optical power coupled to the cladding for initiating the exothermic reaction. Higher ambient humidity and lower ambient temperature will increase the threshold of hydrogen sensor. With the optical heating assisted by a 4-cm-long FBG, the hydrogen sensor can detect 1% hydrogen in air. Further improvements (different LPFGs activating the sensing layer) were reported in 2008 [41]. The sensing probe consisting of 1-cm FBG and 15-dB LPFG displayed greater than 6-nm wavelength shifts when the hydrogen concentration was 4% in volume ratio. The sensor can detect 0.6% hydrogen in air at 25 °C, while the threshold of sensing probe without LPFG was 2.8%. The proposed sensor with best responsibility can detect hydrogen as low as 1.5% even at −50 °C.

**Figure 8:**
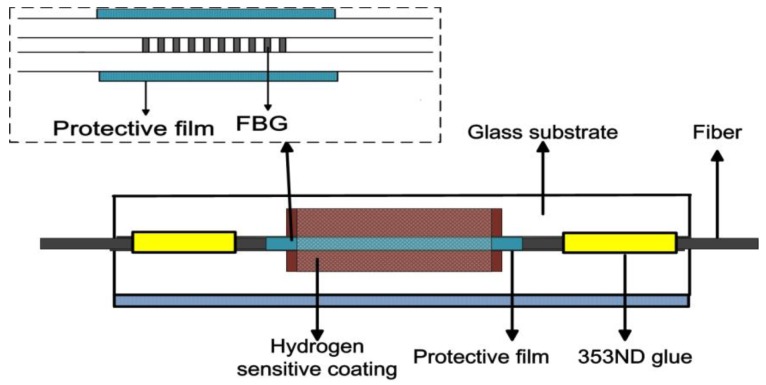
Configuration of FBG hydrogen sensor coated with Pt-loaded WO_3_ coating [43].

Although the reported FBG hydrogen sensor [40,41] has better sensitivity and response rate, its low concentration hydrogen responsibility should be further improved. The energy released by the exothermic reaction should be controlled at proper value so as to ensure its intrinsic safety. An FBG hydrogen sensor based on Pt-loaded WO_3_ material was developed and its sensing characteristics were developed in 2012 by Yang et al. [42]. The performance of the hydrogen sensitive material was enhanced by optimizing its annealing temperature and constitutes. With the continuous effort, the molar ratio of Pt and WO_3_ of hydrogen-sensitive coating was optimized at 1:5 and the annealing temperature was set at 315 °C [43]. Besides, FBG was fixed on glass substrate with higher thermal expansion coefficient so as to increase its temperature sensitivity. Additionally, the glass substrate has a groove for depositing Pt-loaded WO_3_ coatings, which can prevent the shedding of the hydrogen-sensitive coating. The typical structure of sensing probe is shown in Figure 8. At room temperature of 25 °C, FBG hydrogen sensor with best sensitivity has a 448-pm wavelength shift towards 0.8% hydrogen, and the threshold of the FBG hydrogen sensor can reach to 200 ppm in air. Ambient humidity had little effect on its performance. FBG hydrogen sensors showed good selectivity (no response to CO and CH_4_) and repeatability during hydrogen response. Since other reducing gases cannot penetrate the Pt catalyst and react with WO_3_, the Pt-loaded WO_3_ coating has good selectivity towards hydrogen at room temperature. The performance of the sensor at different temperatures was studied in the following work [44]. The hydrogen sensitivity will decrease at lower temperature. However, the hydrogen sensor still can detect hydrogen as low as 400 ppm at 0 °C. Moreover, it is possible to compensate the interference of ambient temperature by using reference FBG. Therefore, FBG hydrogen sensor based on Pt-loaded WO_3_ coating is very promising for distributed hydrogen leakage monitoring in air.

In 2015, Masuzawa et al. [45] developed the fiber grating sensor based on exothermic reaction. Pt/SiO_2_, Pt/WO_3_, Pt/Fe_2_O_3_, Pt/ZnO, Pt/SnO_2_ and Pt/Al_2_O_3_ were fabricated for preparing FBG hydrogen sensors. During hydrogen exposure in switching atmosphere of dry and humid air, Pt/SiO_2_ showed the better stability than other materials. This work proposed the new material for hydrogen sensing. It is worth noting that the stability of the sensor should be further studied due to the obvious different thermal expansion coefficient of Pt and SiO_2_.

**Figure 9:**
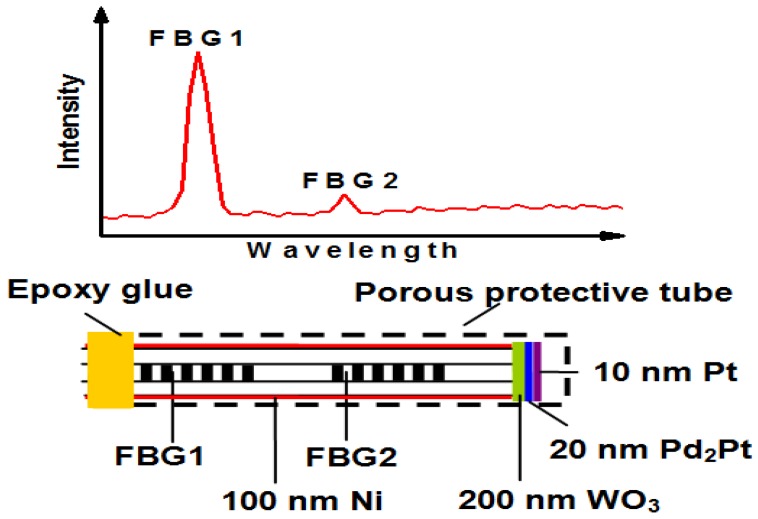
Configuration of high-low Bragg grating with tip coated with WO_3_-Pd_2_Pt-Pt film [47].

WO_3_ film has a good gasochromic effect in hydrogen atmosphere when Pd [50] or Pt [51] is employed as catalyst. A single-mode fiber inscribed with high-low reflective Bragg gratings was deposited with WO_3_-Pd-Pt composite film for hydrogen sensing [42]. Although the performance of sensor is still poor with the employment of a super-luminescent light emitting diode (SLED) light source (80 μW) and an Optical Spectrum Analyzer (AQ6370B, YOKOGAWA, Tokyo, Japan), it proves the good anti-interference for the hydrogen sensor. The performance of sensor is greatly improved by optimizing constitutes and the thickness of the hydrogen-sensitive film as well as writing FBGs (in single mode-fiber) especially at the flat wavelength of the amplified spontaneous emission (ASE) light source [47]. As displayed in Figure 9, the reference and sensing single are transmitted through the same single mode fiber and are detected by same FBG demodulator. Thus, the single noise ratio can be considerably enhanced, and the hydrogen sensor can detect as low as 50 ppm hydrogen in air at an ambient temperature of 25 °C. Several methods, such as optimization of hydrogen-sensitive films, utilization of a more stable ASE light source, and special wavelengths (as sensing intensity) as well as high reflective FBG can be employed to improve the performance of the hydrogen sensor. This work proposes a new method for preparing fiber optic hydrogen sensor with greatly enhanced performance, especially for low concentration hydrogen detection.

In 2016, the long-term stability of Pt-loaded WO_3_ coating was studied by Zhong et al. [52]. With the irradiation of ultraviolet light, the water photolysis effect of Pt-loaded WO_3_ coating can be introduced into the hydrogen sensing process. The experimental tests demonstrate that the hydrogen sensor under UV irradiation exhibits good stability over 3 months. This paper proposes a novel method to enhance the stability of hydrogen sensor based on Pt-loaded WO_3_ coating.

## 4. Challenges for Fiber Grating Hydrogen Sensor

Table 1 and Table 2 list the typical optical fiber grating sensors based on different hydrogen sensitive materials, and their configurations and performances are displayed in these two tables. Meanwhile, some important experimental results of these sensors are shown in the following two tables.

From the above review and discussion, it can be concluded that the performance of fiber grating hydrogen sensors has a close relationship with the hydrogen-sensitive material, configuration of sensing probe and the optical system. The key factor for the fiber grating hydrogen sensor is to prepare stable hydrogen-sensitive material, which can be effectively integrated with the fiber grating. The hydrogen sensors based on pure Pd film easily suffer from the fatal fracture of Pd film caused by its α-β phase transition [12]. By alloying Pd with other metals such as Au [5,6,53], Ni [21,23], Ag [22,24], Cr [25], Mg [54], Y [55], and Pt [56], the structural stability of hydrogen sensitive films can be improved. Due to significant thermal expansion coefficient gap between the silica and Pd-based hydrogen sensitive film [12], an intermediate layer with good adhesion towards silica fiber, such as polyimide [19,20], Ni [21,23], Cr [22] and TiO_2_ [25], should be used to enhance the stability of these hydrogen sensors.

Several other factors, such as operating temperature, ambient humidity and atmosphere, will also affect the performance of the hydrogen sensor. The operating temperature can influence the diffusion rate of hydrogen molecules, hydrogen absorption capability of sensitive materials, and thus affect the sensitivity, response rate and detection threshold of the sensor. Pd tubes [13], Pd [30] and Pd/Ag [34] film display better hydrogen absorption at 23 °C, 30 °C, and 35 °C respectively. When the operating temperatures are increased to 95 °C or 100 °C, hydrogen sensors based on Pd tube and Pd film have quicker response rate and lower sensitivity. The sensor can work normally at room temperature and may not respond to the same concentration hydrogen at low temperatures [16,17]. As for some special facilities, such as liquid hydrogen tanks, the ambient temperature can decrease below 0 °C when hydrogen leaks. Therefore, fiber grating hydrogen sensors, which can give an accurate alarm of leakage at low temperatures, is very crucial to these facilities. The hydrogen sensor with laser heating [17], which can give a quick response toward 1% H_2_ at −150 °C, may be more suitable for these facilities. The operating temperature also affects the performance of an FBG hydrogen sensor based on Pt-loaded WO_3_ coating [40,41,44]. At lower temperature, the proposed FBG hydrogen sensors show the higher threshold [40,41] and poorer sensitivity [44]. Utilizing light heating [16,17] or electric heating device [34], the sensitivity and response rate of the hydrogen sensor can be greatly improved. A similar phenomenon can be observed in the gasochromic effect of Pd/WO_3_ [8]. However, it is not practical for application with utilization of the electric heating device. However, it can be efficient heating equipment for studying the performance of hydrogen sensor at different temperatures. Therefore, employing optical heating system is a promising method to improve the performance of optical hydrogen sensor. Since the performance of the sensor will change at different temperatures, it is necessary to introduce a self-compensation device to keep the hydrogen sensing materials working at the optimized temperature for accurate hydrogen concentration monitoring. Compared to other optical hydrogen sensors, the hydrogen sensors based on fiber grating have a unique advantage to achieve this goal due to their wavelength multiplexing capability. In 2016, two MFBGs were sputtered with Ni film [57], and the sensing MFBG was further deposited with Pd/Ni composite film. This sensor can provide temperature compensation when the sensing probe is heated by heating (980 nm) laser, and the sensing MFBG has obvious wavelength shift when hydrogen is injected in the gas room. The sensing FBG shows better sensitivity and quicker response rate when it is heated by the laser. Although the sensor needs further improvement for application, it demonstrates that a controllable optical heating scheme is feasible.

The second challenge for the optical hydrogen sensor is the interference of the humidity. Most of potential application of hydrogen monitoring is in air. Therefore, developing a hydrogen sensor with good anti-humidity interference is very meaningful. Owing to the water absorption of hydrogen-sensitive material, the sensor based on Pt-loaded WO_3_ coating cannot detect hydrogen below 0.6% under high humidity atmosphere [41]. When exposed to high concentration hydrogen, ambient humidity has little effect on the performance of sensor, which can be attributed to the self-heating effect [58,59] of the sensitive layer. The detection threshold can be greatly reduced by optimizing constitutes of the hydrogen sensitive material [43]. However, the responsibility of these hydrogen may not response to low concentration hydrogen due to the absorption of water molecular when set in air for several weeks. Similar phenomenon can be seen in the gasochromic reaction of WO_3_/Pt [60]. After heating the hydrogen sensitive film at higher temperature, the performance of hydrogen sensor can be recovered. More recently, the sensor stability can be significantly enhanced by ultraviolet irradiation [52], which provides a novel method to overcome the interference of humidity. As for optical fiber hydrogen sensors based on Pd and Pd alloys, the influence of humidity will be much more obvious. In 2008, the hydrogen sensing performance of Pd/Au was studied under room temperature and at different humidity levels [6]. The base line and sensing signal was greatly interrupted by ambient humidity. Since the prepared Pd and Pd alloy are dense and uniform, the surfaces of these films are easily covered with water under a high-humidity atmosphere, resulting in a great deterioration of performance of the hydrogen sensor. By adding polymer layer as protective layer [61,62], the anti-humidity interference of hydrogen sensor can be improved due to their better hydrophobic property. The alternative technique to reduce the humidity interference is heating the sensing material under a proper temperature [6,63], which requires the development of a more reliable optical heating system to meet the demand of application.

The third negative factor of Pd and Pd alloy-based hydrogen sensors is the superficial oxidation of hydrogen sensitive film [14,23]. Freshly prepared sensing probes usually have a quicker response rate and better responsibility to low concentration hydrogen. However, the responsibility will deteriorate after several weeks, especially for low concentration hydrogen. Although Pd is a noble metal, there are several nanometres of PdO on the surface of hydrogen-sensitive film after several months [15,23]. The superficial oxidation film can reduce number of active sites available for the hydrogen, leading to the degradation of the hydrogen sensor. Two methods can be employed to solve this problem. The first method is to prepare Pd/Pt as a hydrogen-sensitive film, which may improve the diffusion rate of hydrogen atom as Pd/Pt can be a highly efficient catalyst material [64]. In addition, depositing Pt [46,47,51,58,59], Au [63] or Pd/Pt [56,64,65] as protective layer may inhibit the oxidization of hydrogen sensitive film, which can ensure the durability of hydrogen-sensitive film.

## 5. Conclusions

**Figure 10:**
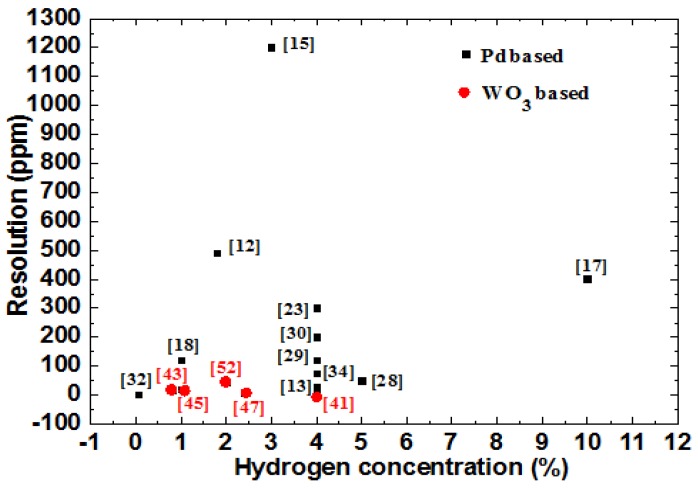
Comparison of the resolution of fiber grating hydrogen sensors based on Pd-based and WO_3_-based hydrogen sensitive materials.

In summary, fiber grating hydrogen sensing systems involve the design of a sensing probe, preparation of hydrogen-sensitive films and acquisition of a sensing signal. Each process is crucial to ensure the performance of the hydrogen sensor. Resolution comparisons of the Pd-based and WO_3_-based hydrogen sensors are shown in Figure 10, and different resolutions (LPFG, Optical Spectrum Analyzer, 20 pm; FBG, FBG demodulator, 1 pm) were used during the calculating process. WO_3_-based hydrogen sensors show higher sensitivity than Pd-based hydrogen sensor (except [13] and [32]). Pd-based hydrogen sensors are more suitable for application in oxygen-free environments, such as in transformer oil [24,25], nuclear natural gas pipelines [62] and waste repositories [66]. WO_3_-based sensors have better performance in air (as shown in Figure 10), so they can be developed for monitoring hydrogen concentration leakage in air. Some obvious improvements of this field were proposed in the last few years. For example, the sensitivity of Pd-based hydrogen sensors [28,32,33,34] was greatly increased, and some of them [32,33] have great potential application in transformer oil. Moreover, the threshold of WO_3_-based fiber grating hydrogen sensor can be reduced to 50 ppm in air at room temperature [47]. Nevertheless, controllable optical heating device can be employed to improve the stability and sensitivity of the sensor [57], which makes it more feasible for accurate hydrogen concentration monitoring. However, preparing fiber grating hydrogen sensor that can be used in a wide range of occasions is still a great challenge. It is much more feasible to develop hydrogen sensor based on different principles for special applications. Meanwhile, it is necessary to consider the above mentioned factors for accurate detection of hydrogen in air. At last, fiber grating sensors are still very promising for hydrogen detection due to their unique characteristics.

## Figures and Tables

**Table 1:** Comparison of fiber grating hydrogen sensors based on Pd and Pd alloys.

Publication Date, Author, Reference	Configuration of Sensing Head, Carrying Gas	Concentration Range, Sensitivity or Wavelength Shift, Response Time, Operating Temperature	Some Important Experimental Results
1999, Sutapun, [12]	560 nm Pd + etched FBG (35 μm), N_2_	0.3%–1.8%, 22 pm/%, -, room temperature	Poor reversibility (H_2_ more than 1.8% (*v/v*)).
1999, Tang, [13]	33 μm Pd tube + FBG, N_2_	4%, 1.4 nm, more than 200 min, 23 °C; 4%, about 2 min, 0.6 nm, 95 °C	Ambient temperature can affect sensitivity and response rate of the sensor
2006, Trouillet, [29]	50 nm Pd + LPFG and FBG, N_2_	4%, 14 pm (FBG), −7 nm (LPFG), less than 2 min, room temperature	LPFG has much better sensitivity
2007, Aleixandre, [15]	5 nm Pd + etched FBG (25 μm), N_2_	0.3%–3.0%, 8.4 pm/% (*v/v*), about 10 min, room temperature	Longer response time after several months due to superficial oxidation
2008, Wei, [30]	70 nm Pd + LPFG, He	4%, −4.3 nm, less than 70 s, 30 °C	Best sensitivity at 30 °C, quicker response rate and lower sensitivity at higher temperatures
2009, Buric, [17]	450 nm Pd + FBG inscribed in 1 cm high attenuation fiber, N_2_	1%–10%, about 37–280 pm, −150 °C (under 1.17-W laser heating)	Greater heating power enables higher sensitivity
2013, Silva, [18]	150 nm Pd + 6 mm FBG inscribed in tapered single-mode fiber SMF (50 μm), N_2_	0.1%–1%, 81.8 pm/%, about 2 min, room temperature	Better sensitivity, without splicing different optical fibers
2014, Dai, [23]	110 nm Pd_91_Ni_9_ + 20 μm FBG fixed on polymer substrate, air	0.5%–4%, about 37 pm/%, 5–6 min, 25 °C	Enhanced sensitivity, superficial oxidation
2015, Jiang, [32]	560 nm Pd/Ag + SPFBG, in transformer oil	100–700 μL/L, 0.477 pm/(μL/L), within 4 h at room temperature, within 1 h at 60 °C	Sensitivity of SPFBG is 11.4 times higher than that of common FBG
2015, Yu, [28]	15 nm Pd + 3.3 μm MFBG, N_2_	−1.08 nm wavelength shift 5%, 60 s, room temperature	Blue wavelength shift, nonlinear response
2015, Karanja, [34]	10 nm Ni + 520 nm Pd_75_Ag_25_ + FBG with microgrooves, air	1%–4%, 5–48 pm, in air, about 60 s, 25 °C; 1%–4%, 50–550 pm, about 50 s, 35 °C	Greatly improved sensitivity at higher temperature

**Table 2:** Comparison of hydrogen sensor based on thermal reaction and gasochromic effect.

Publication Date, Author, Reference	Configuration of Sensing Head, Carrying Gas	Concentration Range, Sensitivity or Wavelength Shift, Response Time, Operating Temperature	Some Important Experiment Results
2008, Caucheteur, [41]	FBG + LPFG, Pt-loaded WO_3_ coating, air	0.6%–4%, 1.2–8 nm, 4 s, 25 °C (FBG + 15 dB LPFG)	Humidity and temperature affect threshold; more active energy enable better responsibility
2014, Dai, [43]	Temperature sensitive + FBG Pt-loaded WO_3_ coating, air	0.02%–0.8%, more than 448 pm; about 2 min, 25 °C.	315 °C annealed Pt:WO_3_ = 1:5 has best sensitivity, threshold of 200 ppm at 25 °C
2015, Masuzawa, [45]	FBG coated with Pt/SiO_2_, Pt/WO_3_, Pt/Fe_2_O_3_, Pt/ZnO, Pt/SnO_2_ and Pt/Al_2_O_3_, air and N_2_	Pt/SiO_2_: 0.1%–1% (in air), about 20–480 pm, less than 20 s, room temperature	Pt/SiO_2_ shows better stability than other materials, poor responsibility in N_2_
2015, Yang, [47]	High-low reflective FBG with tip coated with WO_3_-Pd_2_Pt-Pt film, air	50–23,900 ppm, 10–30 ppm; 20 s, 25 °C	Better sensitivity at low concentration hydrogen, threshold of 50 ppm
2016, Zhong, [52]	Pt-loaded WO_3_ coating + FBG, air	1500–20,000 ppm, about 43.5 ppm; 55–80 s, room temperature	Improved stability with ultraviolet irradiation
